# Teaching Presence vs. Student Perceived Preparedness for Testing in Higher Education Online English Courses During a Global Pandemic? Challenges, Tensions, and Opportunities

**DOI:** 10.3389/fpsyg.2022.891566

**Published:** 2022-05-20

**Authors:** Ronald Morales, Mónica Frenzel, Paula Riquelme Bravo

**Affiliations:** ^1^Departamento de Ingles, Universidad Andres Bello, Santiago, Chile; ^2^Direccion General de Pregrado, Universidad Andres Bello, Santiago, Chile

**Keywords:** online education, English–second language, student preparedness, teaching presence, autonomous learning

## Abstract

In the context of a global pandemic that started in 2020, the Chilean higher education institution Universidad Andrés Bello (UNAB) faced the challenge of giving continuity to its already established blended program for English courses while also starting the implementation of a high-stakes certification assessment for its students using the Test of English for International Communication (TOEIC) Bridge. This study sought to evaluate how much of a mediating factor online teaching presence could be in the context of test preparation within a language course in aspects related to autonomous learning and perceived learning outcomes. A mixed-methods approach was used. It included a survey applied to 1,642 eligible students of the English program. These quantitative data were complemented with students’ comments and teacher interviews. After triangulating quantitative and qualitative data, teaching presence was clearly perceived to be a relevant aspect of the online experience in the studied courses. However, both students’ and teachers’ voices evidenced pervasive challenges and tensions that hinder the potentially transformative benefits that online learning is expected to bring about.

## Introduction

This study is situated in a setting of efforts, challenges, and a worldwide emergency. First, both national and institutional efforts have been made to improve the teaching and learning of English as a foreign language. Globally, there is a need to learn and understand English as it is demanded at every educational level, from primary to tertiary education, due to its status as Lingua Franca and its relevance for industry and the spreading of knowledge (Kabilan et al., 2010). Second, there is an understanding that any educational context set in the twenty-first century can be considered digital education due to the use of networks and learning management systems (Anderson and Rivera-Vargas, 2020), so in that sense, this study also deals with the challenges of online education. Finally, a context of a global pandemic means that Chilean Universities “moved all educational activities to a virtual teaching model using existing institutional software and/or publicly available digital platforms” (Sepulveda-Escobar and Morrison, 2020, p. 587).

In 2012, the Chilean higher education institution Universidad Andrés Bello (UNAB) started implementing a blended approach for the English language program that based its implementation decision on research about online language learning (Johnson and Marsh, 2013). This process has been centered around experiences and evidence which are still the main guidelines for program decisions since then. UNAB included courses for English learning for a total of 500 h over a period of four terms in undergraduate programs. These UNAB students are expected to reach the B1 level according to the Common European Framework of Reference (CEFR) (Universidad Andres Bello, 2016, p. 7). To reach the 500 h committed in the program, a blended model is used to combine activities performed autonomously by students on an online platform and weekly face-to-face practice sessions with the teacher. This is the reason why research on blended learning has been critical in understanding the impact and benefits of this mode for the Department of English (Johnson and Marsh, 2014).

Since 2021, UNAB has decided to use the Test of English for International Communication (TOEIC Bridge) as a benchmark to assess students’ English language performance at the end of the four-term program. This test is particularly useful as it helps assess English skills for work and is used by companies as a reference for hiring (Nae, 2020). The application of this test involved teacher training and preparation for teachers, students, and coordination teams to face this new challenge in the best possible way. Owing to the high stake’s nature of this test, it requires proctoring and should be taken face to face, which was an added challenge to the process due to the context of sanitary restrictions posed by the pandemic.

The UNAB faced the implementation of the TOEIC Bridge in a context where the SARS-CoV-2 (COVID-19) outbreak in 2020 had called for the use of fully online teaching in schools and universities in many countries due to confinement measures (Cancino and Avila, 2021). During 2020 and 2021, English courses at UNAB were taught using a fully online mode of delivery, where the main difference from previous years was the switch to synchronous weekly practice sessions instead of face-to-face meetings between teachers and students. There was also extensive use of the official institutional online platform Blackboard Collaborate to be able to meet the requirements of the academic program (Cancino and Avila, 2021). This change from face-to-face classes to online learning required teachers to acquire new roles and competencies (Atmojo, 2021). Although teachers had had some experience with distance education, in the sense of having used technology mediating the educational experience (Anderson and Rivera-Vargas, 2020), the sudden transition from a bimodal environment into a fully virtual one represented a challenge for everyone involved in the process.

Considering all the challenges mentioned previously, the English Department decided to implement a learning community for teachers in order to concentrate all of its training and preparation efforts on configuring a space of “professional learning.” It is defined as the practice of teachers in support of their pedagogical and content knowledge as well as their teaching practices with the purpose of improving students’ learning and their own relevance in the field (Slagoski, 2019). It is also relevant to mention that various regional studies recognize in teacher preparation a key factor in educational progress (UNESCO, 2013, 2014). Anchored in this new learning community for the teachers of the department, the coordination team organized a series of training activities and webinars centered around the TOEIC Bridge test preparation during the first term of 2021. During these encounters, there was a collective interpretation of the challenges and potential students’ concerns with the new test. Furthermore, teachers collaborated to find the best practices that might have a bigger impact on improving students learning and preparedness (Vaillant, 2019). The intention was to make sure that teachers’ presence in their online courses was seized and perceived by their students.

### Universidad Andrés Bello’s Test of English for International Communication Bridge Test Implementation

As previously mentioned, UNAB has made the teaching and learning of English as one of the core elements within its educational model by including this skill as part of the educational seal which is integral to the graduation profile of students (Universidad Andres Bello, 2016), following a global trend relating to labor market expectations and graduates’ employability (Sandoval and Ormazábal, 2021). The English program for most undergraduate students consists of four courses. During the first term of 2021, students who were enrolled in the 4th and last course of their English program were considered for the first batch of TOEIC Bridge test takers. This first group of students voluntarily registered to take the test face-to-face. As part of the process, the coordination team prepared satisfaction surveys for both teachers and students so they could assess the preparation process. The feedback obtained from those surveys helped identify improvement opportunities and best practices. These were condensed into a document that was socialized with the teaching team. They also informed the implementation for the second term of 2021 when the TOEIC Bridge test was no longer voluntary for students, so preparation and practice became even more relevant. Key aspects, such as mock tests and personal practice material, together with in-class practice material and activities were woven into the program based on the initial feedback from students and teachers. The results of the satisfaction survey for the second term are presented in this report and are also aimed at informing future implementation procedures, consolidating an iterative model of implementation that incorporates feedback in a loop of continuous improvement.

### Self-Regulated Learning

Increasing student motivation through autonomy in learning, the effective use of digital tools and rapport between students and teachers are some of the most relevant and researched aspects of the online education experience (Rivera-Vargas et al., 2021). Therefore, the institution decided to focus its initial efforts on empowering teachers so they could eventually prepare and practice with their own students to face the TOEIC Bridge test with higher levels of confidence by “inserting strategies that encourage the student to work autonomously” (Rivera-Vargas et al., 2021, p. 3371). These strategies can be identified within the self-regulated learning framework, where students use metacognition abilities to think proactively, perform, and self-reflect (Dignath and Büttner, 2008; Ergen and Kanadli, 2017). This framework is defined as an active and constructive process in which individuals set their own learning goals, regulate their cognition, motivation, and behaviors, and are directed and limited by their own goals and contextual features (Pintrich, 2000). Throughout the years, there have been different self-regulated learning models, but most of them have forethought, performance, and self-reflection as major components (Carter et al., 2020). The current study will focus on the performance component, which is related to the strategies that students use to help their own learning and stay focused on tasks, and the self-reflection component, which is related to students reflecting on their performance and evaluating the learning process and outcome (Carter et al., 2020).

### Teaching Presence

Considering the emphasis on teacher preparation, another theoretical framework of relevance for this study is the Community of Inquiry (CoI), which is a collaborative constructivist model of online learning processes that helps inform research and practice (Garrison et al., 2010). This model is comprised of three critical components, namely, cognitive presence, social presence, and teaching presence. This research focuses on teaching presence, which is defined as “the design, facilitation, and direction of cognitive and social processes for the purpose of realizing personally meaningful and educationally worthwhile learning outcomes” (Anderson et al., 2001, p. 5), which involves the organization of content, planning, and instructional guide of an online course. According to previous research, courses with higher levels of teaching presence can provide clearer course structure and more relevant contents that could eventually help students achieve higher efficiency and better learning outcomes (Garrison et al., 2010; Garrison, 2017). Teaching presence can also significantly predict students’ satisfaction (Akyol and Garrison, 2008; Arbaugh, 2008; Maddrell, 2011; Miller et al., 2014; Kyei-Blankson et al., 2016; Yang et al., 2016; Lim, 2018; Shea et al., 2019) and perceived learning in online courses (Shea et al., 2003, 2019; Akyol and Garrison, 2008; Arbaugh, 2008). It is relevant throughout the implementation of an online course, from the design and facilitation to the content knowledge communication, and it has been conceptualized by three subdimensions, namely, instructional design and organization, facilitating discourse, and direct instruction (Caskurlu et al., 2020).

This study aims to assess how much of a mediating factor teaching presence can be when confronted with self-regulated learning components of performance and self-reflection, both understood as indicators of perceived learning, which is also a valid indicator of learning outcomes (Shea et al., 2019). These relationships become relevant considering that the relationship between teaching presence and learning outcomes and their interactions is a valuable research focus (Zhang et al., 2022). There have been studies that have investigated the link between teaching presence and perceived learning, but findings have been inconsistent. This may be due to the possibility that the relationship between teaching presence and perceived learning could be moderated by factors related to context, course types, and other individual factors (Zhang et al., 2022).

In this study, we aim to answer the following question: Is teaching presence a mediating factor to assess students’ perceived learning outcomes when preparing for an international exam to certify their level of English in an online course?

In relation to the guiding question, it is important to clarify that *perceived learning outcomes* refer to the dimensions of performance and self-reflection that are present in self-regulated learning models (Carter et al., 2020).

## Method

The study was carried out using a mixed-methods approach. It implies the use of quantitative and qualitative data gathering strategies in different phases of the study (Pereira-Pérez, 2011) and, in this way, a more accurate perspective of the phenomenon can be obtained, making our perceptions more complete, holistic, and integral (Hernández et al., 2006; Riazi and Candlin, 2014).

In the first phase of the study, a Likert-type scale questionnaire was used as a quantitative data gathering instrument. The construction of this scale was based on the elements of the CoI survey that are related to teaching presence (Anderson et al., 2001). The instrument constructed for this study included three dimensions, namely, teaching presence, performance, and self-reflection. The instrument included seven statements that were related and aligned to each of these dimensions. Once it was designed, the instrument was validated by an international expert in the teaching and learning of English in online courses.

The first four items (Table 1) were used to assess the aspect of teaching presence from the CoI framework. Items 1 and 4 were aligned with statements 1–3, respectively, from the CoI survey, which are part of the subdimension *Design and Organization* of the CoI instrument. Item 2 was aligned with item 11 of the CoI survey, which is part of the *Direct Instruction* subdimension, and item 3 was aligned with item 6 of the CoI survey, which is part of the *Facilitation* subdimension (Anderson et al., 2001).

**TABLE 1:** Survey item alignment and teaching presence.

N°	CoI survey items	Present study survey items	
1	*Design and Organization* 1. The instructor clearly communicated important course topics. 2. 2. The instructor clearly communicated important course goals.	1. I received timely information about the structure and objectives of the TOEIC Bridge exam.	Teaching presence
2	*Direct Instruction* 11. The instructor helped to focus discussion on relevant issues in a way that helped me to learn.	2. I received the information during the course about the TOEIC Bridge exam and I understood the question types and sections it comprises.	
3	*Facilitation* 6. The instructor was helpful in guiding the class toward understanding course topics in a way that helped me clarify my thinking.	3. I received the information during the course about the TOEIC Bridge exam and I understood the benefits of taking it	
4	*Design and Organization* 3. The instructor provided clear instructions on how to participate in course learning activities.	4. I had instances of practice and preparation during some sessions of my English IV course.	

Items 5–7 were used to evaluate the aspects of self-regulated learning. More specifically, statements 5 and 6 assessed performance, and item 7 assessed self-reflection, by means of which students were able to evaluate their perceived preparedness to face the TOEIC Bridge test (Table 2).

**TABLE 2:** Survey alignment and self-regulated learning.

N°	Present study survey items	Self-regulated learning (SRL)
5	I had access to and used the complementary practice and preparation material like the TOEIC Skill-building course available on the English Discoveries Platform.	SRL–Performance
6	It seemed to me that the complementary material was appropriate in my preparation for the TOEIC Bridge exam.	
7	I felt prepared to take the TOEIC Bridge exam.	SRL–Self reflection

### Sample and Participants

The instrument was applied to 1,642 students enrolled in the last course of the English program for undergraduates. This figure corresponds to the total number of students eligible for taking the test. A total of 387 students responded to the survey, which corresponds to a margin error of 4.36 with a confidence level of 95% (Netquest, 2022)^1^. *Microsoft Forms*^2^ was used to set up the instrument and 2 weekly reminders were sent to students to encourage in participation. Results were collected in the corresponding spreadsheet linked to the form and then tabulated to get percentage data for each item and for each of the 5-point scale elements.

### Students’ and Teachers’ Voices

In the second phase of the study, a semi-structured interview was constructed and applied with four teachers who participated in the study. This interview was validated by an international expert in educational research. To supplement the data obtained from the Likert-type scale administered to students, the instrument included an optional open comments section that gathered complementary information about their thoughts on the preparation process for taking the TOEIC Bridge test. Regarding the interviews, both the interviewer and interviewees had an active role in the interaction since they have shared the qualities that are relevant to the study, such as (a) being teachers of the English Department of the institution and (b) having taught the courses which prepared the students for taking the TOEIC test during 2021. The topics of the semi-structured interviews for teachers were aligned with the items from the student Likert-type scale instrument, and they were presented and used during the interviews (Table 3).

**TABLE 3:** Teacher interview guidelines.

Topics	Semi-structured interview guidelines
Topic 1: Teaching presence	About design and organization of the course
	1. Were you able to communicate relevant aspects of the course related with the TOEIC Bridge test preparation (objectives and structure)? How?
	2. Were you able to use the complementary preparation material like the TOEIC Skill-building Course? How was the experience with your students?
	About Facilitation and Direct Instruction
	1. Were you able to explain to students the structure and benefits of taking the TOEIC Bridge test? How?
Topic 2: Self-regulated learning	About performance
	1. Were your students able to access and practice with the complementary preparation material? What was their assessment of this material?
	About self-reflection
	1. Do you think students felt prepared for taking the TOEIC Bridge test? Why?

Apart from these questions, the four interviewed teachers were also able to add any further comments about the process of implementation, preparation, and application of the test. When presenting teachers’ quotes, they are followed by a T and a designated number for individualization purposes. The same principle was used when presenting students’ quotes, which are followed by an S and a designated number.

In the third phase of the study, qualitative and quantitative data were triangulated to generate preliminary conclusions that were derived from the discussion and conclusion of the study.

## Results

The results are presented by triangulating the quantitative data from the survey and the voices of the students who emerge from their written opinions and those of the teachers who emerge from the semi-structured interviews conducted.

The analysis will be presented and organized along the dimensions of teaching presence, self-regulated learning, performance, and self-reflection. A preliminary synthesis will be presented at the end of each section.

### Teaching Presence

Understanding that teaching presence implies a teacher who facilitates and makes use of direct instructional methods to share content knowledge with students throughout an online course (Caskurlu et al., 2020), it can be seen that students identify it specifically in *Direct Instruction* (question 2 of the survey), which shows a level of agreement of 86% among those who were consulted (Table 4).

**TABLE 4:** Survey results for teaching presence.

Teaching presence							

Questions	Total N°	Strongly agree %	Agree %	Neutral %	Disagree %	Strongly disagree %	Total %
I received timely information about the structure and objectives of the TOEIC Bridge exam.	387	58%	30%	6%	2%	3%	100%
I received the information during the course about the TOEIC Bridge exam and I understood the question types and sections it comprises.	387	63%	26%	6%	1%	4%	100%
I received the information during the course about the TOEIC Bridge exam and I understood the benefits of taking it	387	55%	28%	11%	3%	3%	100%
I had instances of practice and preparation during some sessions of my English IV course.	387	61%	25%	8%	2%	5%	100%
Average		59%	27%	8%	2%	4%	

On a more specific analysis, we found a relationship between the students’ perception of the question *I had instances of practice and preparation during some sessions of my English IV course*, in which 85% agreed, with the following comments written in the surveys related to the teacher’s preparatory role for the exam:

“Our teacher prepared us very well and I was happy with my performance” (S1).

“The teacher answered all doubts and was very kind and accurate to comment about this exam” (S13).

“My teacher was excellent. Having done other certification tests in the past, the teachers would scare us talking about its difficulty and importance, but our teacher was very emphatic and prepared us a lot, but without exaggerating about the test” (S5).

Another group of quotes shows that students have a high appreciation of their teachers and different strategies used to prepare them for the exam. Among these strategies, students highlight reminding, reinforcement, and the use of the platform tools, such as announcements and synchronous sessions, which are stated in the following excerpts:

“It was entertaining to prepare it each class, which made me be much more prepared to take this important test…My teacher was very good, which enhanced my learning even further” (S8).

“Our teacher prepared us well…she indicated its importance both in online classes and in the announcements” (S22).

In the same way, the teachers consulted agree with the students’ assessment and perception of their presence as a support in the preparation process for the exam, as can be seen in the following quotations:

“I emphasized the relevance of the test for their future” […] “When I received a mail with a question from a student, I would send an announcement with the answer as a reference for the whole class” (T2).

“Due to the student’s profile and career I have to adapt the material to cater to their specific needs” (T3).

“There were 3 or 4 weekly announcements on my part” […] “I had to insist” […] “I used apps to complement their work” (T1).

“I received a lot of mails asking about information present in the announcement…I had to use a lot of time repeating information about the test” […] “But I also thought it was important to empathize with students” (T4).

The information from the quotes shows that teachers used platform tools to keep in constant communication with their students, such as announcements. In addition, they developed supplementary materials and used digital resources in addition to the platform resources to ensure that students were practicing their second language skills.

It is important to note that, although most of the students’ opinions are positive, only 18% of the students made written comments, which could suggest that they perceived a lesser presence of their teacher, but not to the extent that they were willing to mention him/her. Within the percentage of the responses, it was possible to identify examples with negative evaluations, as evidenced in the following quotes:

“I thought the TOEIC preparation was insufficient, the preparation units were few and very similar to the conventional units of the course” (S16).

“The test advanced too fast without offering the possibility of repeating the audio…awful experience” (S7).

The selected quotations reveal, on the one hand, that the students do not seem to find the preparation units challenging and, on the other hand, they are not clear about the requirements and formats of the test. The first quote suggests that the students did not really know the structure of the test, which implies having only one opportunity to listen to the recorded audios. The second quote could imply a lack of adaptation of the available materials. Both situations can be related to either the lack of teacher presence or the problems of student interaction with the course content.

Although the comments are aligned with the students’ remarks, the teachers’ perceptions focused mainly on what they did to transmit their knowledge. There was no explicit mention of whether they ensured that the students understood and comprehended the concepts and instructions that were being given. The teaching presence perceived by students, according to Caskurlu et al.’s (2020) meta-analysis, should contribute to the development of metacognition and self-regulation skills by providing “practical insights on how to be actively involved in the course thereby constructing their knowledge through collaboration, interaction with others, and experiencing others’ points of views” (Caskurlu et al., 2020, p. 11). In contrast, the learning community for teachers should analyze the type of response given to students, beyond a reminder and/or suggestion, as this community should support a process of collective learning and an interest in pedagogical strategies (Vaillant, 2019).

Despite the fact that the analysis shows the forms of teacher presence that are effectively perceived by students, it is still incipient, especially because the elements of paradigms that are deeply rooted in the more traditional pedagogies of transmission and direction of learning by the teacher still persist (Charbonneau-Gowdy and Cechova, 2017). This means that despite the efforts at the national and institutional levels, education continues to be perceived only as the dissemination of knowledge and content in virtual learning environments (Anderson and Rivera-Vargas, 2020). This is still far from the constructivist and collaborative process that online learning hopes to develop and contribute to the new professional scenarios, through resources, such as synchrony and the use of various digital resources that strengthen autonomous learning.

### Self-Regulated Learning

#### Performance

In terms of the performance variable, questions 5 and 6 refer to this aspect. Notably, 78% of the students agree that they had access to and used the supplementary materials available on the platform (Table 5). In addition, 89% of the students agree that the supplementary material was appropriate for the preparation of the exam. However, the percentages of the neutral option increase by 11 and 12%, and the percentages of the disagreement options decrease by 10 and 8%) (Table 5).

**TABLE 5:** Survey results for self-regulated learning (performance).

SRL–Performance							

Questions	Total N°	Strongly agree %	Agree %	Neutral %	Disagree %	Strongly disagree %	Total %
I had access to and used the complementary practice and preparation material like the TOEIC Skill-building course available on the English Discoveries Platform.	387	51%	27%	11%	4%	6%	100%
It seemed to me that the complementary material was appropriate in my preparation for the TOEIC Bridge exam.	387	49%	30%	12%	3%	5%	100%
Average		50%	28%	12%	4%	6%	

The perception of the accessibility and usefulness of the test preparation materials are reinforced by the voice of the students in the following quotes:

“Excellent preparation to take the test, the practice material in the platform is very similar to the actual TOEIC test” (S12).

“The preparation is comprehensive, although it requires a lot of time but that can be solved with good organization” (S3).

“In general, very good preparation, although I wish we had more time to practice with the teachers” (S14).

“Excellent way of working, very dynamic and fun” (S15).

However, not all students who responded to this part of the survey have the same opinion as evidenced by the following quotes:

“I thought the TOEIC preparation was insufficient, the preparation units were few and very similar to the conventional units of the course” (S16).

“The practice in class was not the same because they didn’t give us the time constraints demanded in the real TOEIC” (S18).

“I didn’t like the fact that we could listen to questions many times and only once in the actual test. The conditions in the preparation test should be the same as in the real test” (S20).

“Improve practice for the test, maybe create tutorials and specific review sessions for that” (S23).

In contrast, teachers’ voices give us clues as to how they perceive what the students say, and the following quotes give evidence of their perceptions:

“Many students pass the course just because they complete self-paced activities” (T3).

“The number of students engaging with the practice material is low. Many of them work and had to watch the recordings later. Although attendance, to the synchronous sessions, was low, those who participated in sessions really took advantage of them” (T1).

“Students are afraid of making mistakes” […] “We should reward students’ effort” […] “We should help them explore the language and not dwell on grammar jargon too long” (T2).

“TOEIC should be compulsory and needs to be part of the course grades.” […] “Students should perceive this test as an experience” (T4).

To summarize, it can be observed that the students highly value the presence of their teachers regarding their potential performance, both at the level of perception and in an evident way in some of the quotes. However, in contrast, it can be noticed that the students’ voices externalize the responsibility for what happens in the courses in terms of their potential performance in the test. Even when they express themselves negatively, they only mention what happened and not what they could have done together with the teacher to improve their English proficiency and perform better in the exam. The previous point seems to align with the fact that “efforts to increase student engagement remain generally unsuccessful” (Charbonneau-Gowdy, 2018, p. 56).

In contrast, teachers’ voices follow an understanding of the importance of context and the impossibility of bringing the content successfully to any space and to any student (Anderson and Rivera-Vargas, 2020). There is also a perception of poor student performance due to a lack of engagement with the content and attendance problems, which have been exacerbated by the context of confinement and entirely virtual interactions that affected student participation. This has been supported by the reports of absenteeism, no use of cameras and microphones, and a general reluctance on the part of the learners to engage online (Charbonneau-Gowdy et al., 2021).

It is evident when contrasting the discourses that both students and teachers externalize the success or failure of the results on each other. However, it would be interesting to find out how both actors are involved in teaching and its real improvement. In contrast, how students really impact their performance based on their self-regulation and autonomous behaviors when learning. This is particularly relevant in an educational environment that was influenced by COVID-19 restrictions, which changed the way lessons were delivered and the use of online platforms that forced students and teachers to adapt to the new circumstances (Cancino and Avila, 2021). The teaching presence in virtual spaces is understood as the facilitating factor of active environments where teachers and students share ideas and opinions and where social presence is demonstrated through engagement in collaborative discussions (Charbonneau-Gowdy et al., 2021). Clearly, this needs to have an impact on motivation of students so that, regardless of the circumstances, they can make the most of the design of learning activities.

#### Self-Reflection

In this study, self-reflection refers to *the subjective perception of students own preparedness to face the TOEIC Bridge test*. It is interesting to note that in question 7, only 68% of the students agreed with the statement. This shows a 10% decrease in agreement with respect to their perception of their performance in the test and 18% decrease in relation to the assessment of the teaching presence in the test preparation course (Table 6).

**TABLE 6:** Survey results for self-regulated learning (self-reflection).

SRL–Self reflection							

Questions	Total N°	Strongly agree %	Agree %	Neutral %	Disagree %	Strongly disagree %	Total %
I felt prepared to take the TOEIC Bridge exam.	387	40%	28%	22%	4%	5%	100%

Nevertheless, there is evidence of students’ and teachers’ assessment of the preparation of the test, as the following quote shows:

“Preparation for this test was really good […] we knew the dynamic of the test […]I felt prepared in a way, but nervous” (S6).

“Students were enthusiastic about the test, but they didn’t trust their skills” […] “We should start TOEIC preparation from the first course” […] “Students get to course four unprepared” (T2).

In summary, both students’ and teachers’ perceptions point to the possibility that teacher presence alone is not sufficient to ensure that students feel prepared for a major assessment. These comments shed light on some other factors that could affect students’ readiness and may well inform future iterations of implementation. It is also important to note the very low rates of participation in the comment section of the instrument and the willingness of students to share their opinions, which could imply that the teaching presence is still insufficient in making students aware of their role in the learning process.

We should also consider that social presence and having high expectations of students’ learning, independent of their socio-cultural status, can be directly related to students’ performance in the exam. Due to this, “Further ethnographic, social-based research […] is at the heart of uncovering the contextual issues that are unique to each setting” (Charbonneau-Gowdy, 2018, p. 65).

## Discussion and Conclusion

We started this report by setting the study in a context of efforts, challenges, and a worldwide emergency. As an initial discussion point, we can focus on the efforts made by the institution to empower its team of English teachers by means of a virtual learning community. This community helped prepare teachers and tried to make sure their presence was perceived by their respective students while preparing for the TOEIC test. Although limited to this institution, the survey results seem to confirm that these efforts are headed in the right direction, since students indicated high levels of agreement in all subdimensions with direct instruction evidencing the highest percentage. These results confirm the importance of considering teaching presence when designing and implementing online courses (Caskurlu et al., 2020). However, these results complemented with our qualitative data also suggest and may confirm that contextual factors are important in “how teaching presence impacts perceived learning, and how individual factors moderate the association” (Zhang et al., 2022, p. 9).

Second, we now know that the worldwide emergency of the COVID-19 pandemic brought new challenges to teaching and learning for students and teachers alike. These challenges resonate with the lingering tensions between what is needed to support learners and what it means to provide enough guidance, structure, and support in an online course (Carter et al., 2020). Qualitative data from the teacher interviews and students’ comments attest to the relevance of teachers’ role in helping students become more self-regulated learners. In that sense, we agree with the importance of teachers using metacognitive strategies to increase students’ achievement and self-regulation in different learning environments (Ergen and Kanadli, 2017).

Students’ comments on performance also show that teaching strategies need to be consistent and discussed within the learning community of teachers if the goal of improving learning and preparedness is to be met (Vaillant, 2019), especially considering the iterative model of implementation of preparation and practice for the TOEIC Bridge test and the updates on best practices that it proposes. Although teaching presence was perceived by students in this study and confirmed by some of the comments of both students and teachers, it is still unclear if it influenced performance since the interviewed teachers expressed concerns regarding students’ attendance and engagement in the context of online teaching and learning (Charbonneau-Gowdy, 2018).

It was interesting to notice that the perceived levels of teaching presence did not necessarily translate into higher levels of perceived preparedness. Furthermore, Teacher 2’s remarks hint at the issues of confidence in their own skills and issues with planning the preparation of a course. They even hint at possible issues with placement instruments and assessments that allow students to reach the last course “unprepared.” These issues, although relevant and enlightening for future research, should make us reflect on the actual ways in which teachers and students are interacting in online courses and how technology is being used. This is especially relevant if we aim to truly transform student–teacher interactions and roles to finally reach the original promises of digital technologies for a more connected and collaborative learning experience (Anderson and Rivera-Vargas, 2020). We have probably reached a point where we should finally focus on aligning theory and practice on online learning to find solutions that could go beyond the challenges posed by the pandemic (Charbonneau-Gowdy et al., 2021).

When confronting the guiding question of this study *Is teaching presence a mediating factor to assess students*’ *perceived learning outcomes when preparing for an international exam to certify their level of English in an online course?* with the results obtained through the different instruments applied, we can see an alignment between the survey results, and students’ and teachers’ comments about the strategies and methodologies used during preparation for the TOEIC Bridge test. These results seem to be in line with other studies that indicate that teaching presence predicts student satisfaction (Akyol and Garrison, 2008; Arbaugh, 2008; Maddrell, 2011; Miller et al., 2014; Kyei-Blankson et al., 2016; Yang et al., 2016; Lim, 2018; Shea et al., 2019). When incorporating qualitative data, we realize that perceived teaching presence does not necessarily equate to increased performance and self-reflection (understood in this study as *perceived learning outcomes*). On the contrary, it hints at deeper issues that are related to how the expectations of online learning pushing toward more constructivist ways of teaching and learning have yet not been reflected in reality (Anderson and Rivera-Vargas, 2020). In light of these findings, we agree with the need to apply grounded research to put into practice innovations and new instructional design models which should be based on contemporary e-learning theories (Charbonneau-Gowdy et al., 2021). This is particularly relevant considering that low attendance and engagement have been commonplace since the pandemic started in 2020, with online classes being the only viable option for some time to ensure continuity in the institution’s academic activities (Cancino and Avila, 2021).

Considering the limitations of this study, we can first point out that it was carried out with students studying in one semester. Future studies could be carried out to compare the results of students over two or more semesters to identify potential differences among levels. Second, the number of teachers interviewed was four, so future studies including more teachers could generate further insights into the challenges they face when dealing with online courses. Although students’ voices were included through their comments, a future study could consider interviews and focus groups that could produce richer perceptions about their experience. Finally, students’ results in the TOEIC Bridge test were not considered. In a future study, they could be included to enrich the analysis and triangulation of quantitative and qualitative data from students and teachers about their perceptions of exam preparation.

With the expected and gradual return to normal face-to-face classes in 2022 in Chile, future research on the subject could analyze the same aspects of teaching presence and self-regulated learning. The aim would be to identify potential differences that online and face-to-face exam preparation may have on students’ and teachers’ perceptions. Considering Charbonneau-Gowdy et al.’s (2021) suggestions, it would also be interesting to explore the potential impact that different methodologies, such as “project-based learning,” could have on the interaction between students and teachers within online language courses in higher education.

## Data Availability Statement

The raw data supporting the conclusions of this article will be made available by the authors, without undue reservation.

## Author Contributions

RM worked on surveys, analysis, and writing of the manuscript. MF and PB worked on reviewing and proofreading of drafts. All authors contributed to the article and approved the submitted version.

## Conflict of Interest

The authors declare that the research was conducted in the absence of any commercial or financial relationships that could be construed as a potential conflict of interest.

## Publisher’s Note

All claims expressed in this article are solely those of the authors and do not necessarily represent those of their affiliated organizations, or those of the publisher, the editors and the reviewers. Any product that may be evaluated in this article, or claim that may be made by its manufacturer, is not guaranteed or endorsed by the publisher.

## Footnotes

1
shorturl.at/dfvJN

2
https://forms.office.com/r/Bbst5cy1iP
